# A Case of Spontaneous Pneumomediastinum with Subcutaneous Emphysema in Children

**DOI:** 10.3390/children5020022

**Published:** 2018-02-07

**Authors:** Said Benlamkaddem, Mohamed Adnane Berdai, Smael Labib, Mustapha Harandou

**Affiliations:** Maternal and Paediatric Critical Care Unit, Hassan II University Hospital, 1835 Fez, Morocco; adnane.berdai@yahoo.fr (M.A.B.); smael.labib@usmba.ac.ma (S.L.); harandoumustapha@yahoo.fr (M.H.)

**Keywords:** emphysema, pneumomediastinum, pneumopericardium, Valsalva’s maneuver

## Abstract

Spontaneous pneumomediastinum is defined as free air or gas contained within the mediastinum, which almost invariably originates from the alveolar space or the conducting airways. It is rare in pediatric patients; however, occasional cases are reported to result from forced Valsalva’s maneuver due to cough, emesis, a first attack of wheeze, or asthma exacerbations. We report the case of a 7-year-old previously healthy girl, with a history of persistent dry cough one day before, who was brought to our unit with face, neck and chest swelling. The chest X-ray and computed tomography (CT) scan showed subcutaneous emphysema with pneumomediastinum and pneumopericardium without evidence of the origin of this air leak. Laboratory tests and the bronchoscopy were normal. The patient was admitted in the pediatric critical care and received noninvasive monitoring, analgesia, oxygen, and omeprazole as a prophylaxis for a gastric ulcer. The patient improved, subcutaneous emphysema resolved, and she was discharged on the third day.

## 1. Introduction

Spontaneous subcutaneous emphysema and pneumomediastinum refer to the presence of gas in soft tissue of the neck, thorax and the mediastinal space [1]. It is a separate entity that occurs in previously healthy individuals without underlying respiratory diseases resulting in an increase in intrathoracic pressure. Spontaneous pneumomediastinum (SPM) is an uncommon disorder with an incidence of 0.0025% among emergency room visits [2,3], in whom it primarily appears as a complication of thoracic injury, surgical operation, or pulmonary infection [4]. 

## 2. Case Presentation

A 7-year-old previously healthy girl, with a history of persistent dry cough one day before due to a rhinopharyngitis, was brought to the emergency department of maternal and pediatric hospital of Hassan II university hospital, with face, neck and chest swelling responsible for a headache, sore throat and neck pain. 

On initial presentation, the girl was slightly short of breath with good arterial saturation (spO_2_ 98%), her blood pressure and heart rate were normal. She had face, neck and chest swelling with crepitation during palpation. During cardiac auscultation there were, also, reduced heart sounds. We note that there were no inflammation signs (erythema and local heat). 

**Figure 1:**
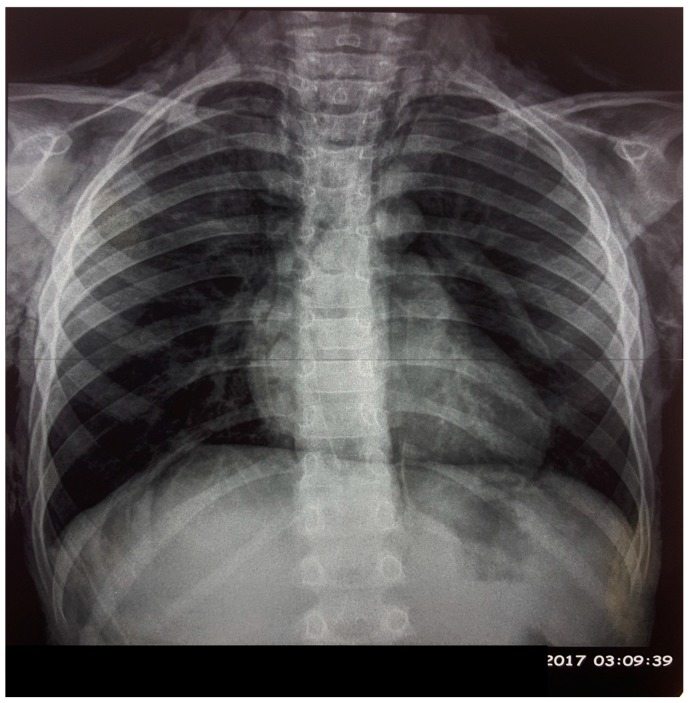
Chest X-ray showing emphysema and pneumomediastinum.

**Figure 2:**
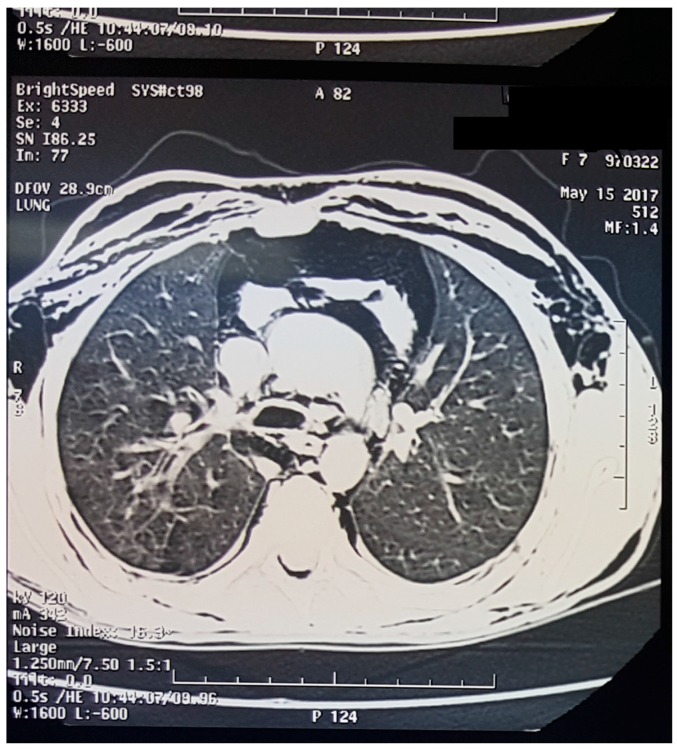
Computed tomography (CT) scan showing pneumomediastinum, pneumopericardium and emphysema.

The chest X-ray (Figure 1) showed subcutaneous emphysema with pneumomediastinum. The computed tomography (CT) scan (Figure 2) demonstrated, in addition to the findings in chest X-ray, a pneumopericardium without evidence of the origin of this air leak. 

Laboratory tests: arterial blood gas: pH: 7.46, paO_2_ 95 mmHg, paCO_2_ 35 mmHg, bicarbonate 24.2 mmol/L. Biochemistry: C reactive protein 14 mg/L; normal renal function tests and electrolyte levels. Hematology: hemoglobin 11.5 g/dL; white blood cell count 12.7 × 10^9^ cells/L; platelet count 245 × 10^9^ cells/L. The bronchoscopy, which was performed one day later, was normal. 

The patient was admitted to pediatric critical care and received noninvasive monitoring, analgesia, oxygen (5 L/min), and omeprazole as a prophylaxis for gastric ulcer.

Over of the next few days she improved, subcutaneous emphysema resolved, and she was discharged on the third day. 

## 3. Discussion

Spontaneous pneumomediastinum is defined as free air or gas contained within the mediastinum, which almost always originates from the alveolar space or the conducting airways [5,6,7,8,9]. The most frequent underlying factor is alveolar rupture caused by overdistension or increased alveolar pressure. Alveolar rupture allows bubbles of gas to disseminate along the pulmonary vasculature toward the hilum and mediastinum and subsequently to the soft tissue of the cervical region through fascial planes connecting these areas [10]. In pure pneumomediastinum, the parietal pleura remain intact.

SPM is a rare condition seen primarily in older children and adolescents, with a first peak in incidence between 6 months and 3 years of age due to respiratory infections; however, occasional cases are reported to result from forced Valsalva’s maneuver due to cough, emesis, a first attack of wheeze, or asthma exacerbations [11,12,13,14]. 

Chest pain and dyspnea are the most common initial symptoms. Head and neck manifestations are always secondary to pneumomediastinum, but they represent the first warning of mediastinal emphysema, caused by air accumulation subsequently disseminating along the fascia to the cervical region [15]. The primary symptom among patients with SPM in the literature review is visible neck swelling with dull pain but no inflammation, such as redness, erythema, or local heat. During the palpation examination, observation of crepitation over the neck was consistent with subcutaneous emphysema. Other frequently reported symptoms were cough, voice change and dysphagia.

Less-common findings on physical examination in patients with SPM are crepitations occurring with reduced heart sound (Hamman’s sign) on pericardial auscultation. The diagnosis of SPM is made based on radiologic findings [16].

Chest radiography may not always be sufficient to make the diagnosis of SPM. Chest CT provides confirmation of the diagnosis, as well as assessment of any associated causes or abnormalities and to exclude life-threatening differential diagnoses. A gastrointestinal (GI) workup (e.g., esophagography) should be reserved for patients with significant GI symptoms, such as those with Boerhaave syndrome, which is associated with high mortality and morbidity. 

SPM generally follows a benign and self-limiting course, and the usual treatment is bed rest, analgesics, and oxygen therapy. Breathing pure oxygen has been advocated to reduce the partial pressure of nitrogen in the subcutaneous air, which allows acceleration of its resorption. Severe cases, such as tension pneumomediastinum or tension pneumothorax, may require invasive measures. Underlying disorders should be treated, if possible [17,18].

## 4. Conclusions

SPM is a rare benign entity. Patients may be seen with symptoms predominantly in the head and neck region. Important and potentially life-threatening differential diagnoses must be excluded using appropriate investigations.
